# A novel model for predicting a composite outcome of major complications after valve surgery

**DOI:** 10.3389/fcvm.2023.1132428

**Published:** 2023-05-17

**Authors:** Zhenzhen Cheng, Yishun Wang, Jing Liu, Yue Ming, Yuanyuan Yao, Zhong Wu, Yingqiang Guo, Lei Du, Min Yan

**Affiliations:** ^1^ Department of Anesthesiology, The Second Affiliated Hospital of Zhejiang University School of Medicine, Hangzhou, China; ^2^ Department of Anesthesiology, West China Hospital of Sichuan University, Chengdu, China; ^3^ Department of Cardiovascular Surgery of West China Hospital, Sichuan University, Chengdu, China

**Keywords:** prediction model, composite major complications, all-cause mortality, stroke, myocardial infarction, severe acute kidney injury

## Abstract

**Background:**

On-pump valve surgeries are associated with high morbidity and mortality. The present study aimed to reliably predict a composite outcome of postoperative complications using a minimum of easily accessible clinical parameters.

**Methods:**

A total of 7,441 patients who underwent valve surgery were retrospectively analyzed. Data for 6,220 patients at West China Hospital of Sichuan University were used to develop a predictive model, which was validated using data from 1,221 patients at the Second Affiliated Hospital of Zhejiang University School of Medicine. The primary outcome was a composite of major complications: all-cause death in hospital, stroke, myocardial infarction, and severe acute kidney injury. The predictive model was constructed using the least absolute shrinkage and selection operator as well as multivariable logistic regression. The model was assessed in terms of the areas under receiver operating characteristic curves, calibration, and decision curve analysis.

**Results:**

The primary outcome occurred in 129 patients (2.1%) in the development cohort and 71 (5.8%) in the validation cohort. Six variables were retained in the predictive model: New York Heart Association class, diabetes, glucose, blood urea nitrogen, operation time, and red blood cell transfusion during surgery. The C-statistics were 0.735 (95% CI, 0.686–0.784) in the development cohort and 0.761 (95% CI, 0.694–0.828) in the validation cohort. For both cohorts, calibration plots showed good agreement between predicted and actual observations, and ecision curve analysis showed clinical usefulness. In contrast, the well-established SinoSCORE did not accurately predict the primary outcome in either cohort.

**Conclusions:**

This predictive nomogram based on six easily accessible variables may serve as an “early warning” system to identify patients at high risk of major complications after valve surgery.

**Clinical Trial Registration:**

[www.ClinicalTrials.gov], identifier [NCT04476134].

## Introduction

Annually, approximately 275 000 heart valve surgeries involving cardiopulmonary bypass (CPB) are carried out worldwide to treat symptomatic valve disease (1), and more than 20 000 of those surgeries end with patient morbidity or mortality (2, 3). The rate of mortality after cardiac surgery varies from 1.0% to 8.6% (2), and rates are also high for stroke (2.2%), myocardial infarction (1.5%) and severe acute kidney injury (1.3%) (4–6). Early identification of patients who are at risk of major complications may improve their management and prognosis after valve surgery.

Several models have been constructed to predict the risk of complications after cardiac surgery (7–13). Most of them (9–11), such as the Sino System for Coronary Operative Risk Evaluation (SinoSCORE), were developed to predict a specific adverse event after surgery based on coronary artery bypass grafting (CABG). Classical models, such as the scoring systems of the Society of Thoracic Surgeons (STS) and the European System for Cardiac Operative Risk Evaluation II (EuroSCORE II), were developed from data from patients in the West and involve many predictors, making them difficult to apply in the clinic (7, 8, 13).

The present study aimed to develop a predictive model that was based on Asian patients and that involved a relatively small number of easily accessible clinical parameters. The model exploits the fact that the various adverse events after valve surgery share similar risk factors, such as diabetes, New York Heart Association (NYHA) class, use of CPB, operation time and need for transfusion (14–16). This overlap in risk factors probably reflects that many of these events share the same causes of poor organ perfusion and systemic inflammation induced by CPB (17–19). Therefore, when developing the model, we designed it to predict not one specific adverse event but a composite outcome of major complications.

## Materials and methods

This study was approved by the Ethics Committees of the Second Affiliated Hospital of Zhejiang University School of Medicine in Hangzhou (096/2017) and West China Hospital of Sichuan University in Chengdu (256/2017). The requirement for informed consent was waived due to the retrospective nature of this study. The present study took its data from a previously described dataset that we have previously analyzed for other purposes (16, 20, 21).

### Participants

To be enrolled in the present study, patients in our database had been at least 18 years old, scheduled for valve surgery under CPB with or without CABG from January 1, 2011 to June 30, 2017 at West China Hospital, or from September 1, 2013 to June 30, 2017 at the Second Affiliated Hospital of Zhejiang University School of Medicine. Patients were excluded if they (1) underwent emergency surgery, (2) died in the operation room, (3) could not be weaned off CPB, or (4) underwent combined surgery involving aortic replacement or tumor resection. Two investigators who were blind to the study hypothesis used a predefined form to collect relevant data from the database.

### Endpoint

The primary outcome was a composite of the following major complications: (1) all-cause mortality (22), (2) myocardial infarction (22), (3) stroke (23), and (4) severe acute kidney injury (24). All major complications were defined as those occurring new after surgery and during hospitalization. Detailed definitions are provided in Supplementary Table S1.

### Data collection

Data were extracted from the database relevant to variables that we considered potential predictors of the composite primary outcome, based on general clinical practice, transfusion-related adverse outcomes after cardiac surgery (21, 25), risk factors of acute kidney injury (16) and other research (8, 9, 26). Variables included age, sex, and ethnicity; body max index (BMI), current smoking, current alcohol consumption, comorbidities and medical history; American Society of Anesthesiologists (ASA) physical status; NYHA classification; pre-operative medication and laboratory findings; type of surgery and intra-operative data. More details are provided in Supplementary Table S1.

Potential predictors were excluded if data for them were missing for >10% of patients. When data were missing for no more than 10% of patients, multiple imputation based on five replications was performed.

### Statistical analysis

Data were analyzed using R 4.1.0 (https://www.r-project.org). Continuous data were tested for normality and presented as mean (SD) if normally distributed, or as median (IQR) if skewed. Differences between the development and validation groups were assessed for significance using the Mann–Whitney *U* test, Student's *t* test, or Welch's *t* test as appropriate. Categorical data were reported as number (proportion), and intergroup differences were assessed using Fisher's exact test or the chi-squared test.

To reduce the risk of over-fitting in the predictive model, potential predictors were selected using least absolute shrinkage and selection operator (LASSO) regression. Ten-fold cross-validation was used to determine the penalty parameter (*λ*), which was optimized based on the criterion of one standard error away from the minimum binomial deviance. Variables with non-zero coefficients were entered into multivariate logistic regression, the results of which were expressed as odds ratios (ORs) and 95% CIs.

Based on the weight of each predictor in the model, a nomogram was drawn. The performance of the model was assessed as recommended (27) in terms of the the areas under receiver operating characteristic curves (AUC) to discriminate patients who did or did not experience the composite primary outcome, the calibration curve and accompanying Brier score, and the net benefit as determined using decision curve analysis. AUC was corrected using bootstrapping (1,000 replications). These performance assessments were carried out for the development cohort from West China Hospital and for the validation cohort from the Second Affiliated Hospital of Zhejiang University School of Medicine. To benchmark our model's performance, we compared it to the performance of SinoSCORE against both cohorts.

## Results

A total of 7,696 patients who underwent valve surgery at the two study sites were screened for enrollment, of whom 255 patients were excluded because they underwent emergency surgery (10), died during the operation (19), could not be weaned off CPB (4), or underwent a combination of surgical procedures also involving ascending aortic replacement or tumor resection (222). More details are shown in Supplementary Figure S1.

In the end, 7,441 patients were included in the final analysis, comprising 6,220 in the development cohort and 1,221 in the validation cohort. The two groups were comparable in most baseline and intraoperative parameters (Table 1, Supplementary Table S2). Compared to the validation group, patients in the development group were significantly older (50 ± 11 vs. 56 ± 11 years), more likely to be women (55.5% vs. 50.5%), more likely to undergo multi-valve surgery (55.2% vs. 41.8%), and less likely to undergo a combination of valve surgery and CABG (1.1% vs. 6.6%).

**Table 1:** Baseline characteristics of patients in the development and validation cohorts.

Characteristic	Overall (*n* = 7,441)	Development cohort (*n* = 6,220)	Validation cohort (*n* = 1,221)	*P*-value
Baseline characteristics				
Age, years	51 (10.9)	50 (11)	56 (11)	<0.001
Female	4,571 (61.4)	3,449 (55.5)	599 (50.9)	<0.001
Ethnicity				<0.001
Han Chinese	7,256 (97.5)	6,039 (97.1)	1,217 (99.7)	
Tibetan	96 (1.3)	94 (1.5)	2 (0.2)	
Other	89 (1.2)	87 (1.4)	2 (0.2)	
BMI, kg.m^2^	22.78 (3.18)	22.85 (3.18)	22.43 (3.19)	<0.001
Current alcohol use	1,294 (17.4)	1,097 (17.6)	197 (16.1)	0.215
Current smoking	1,878 (25.2)	1,520 (24.4)	358 (29.3)	<0.001
Comorbidities and medical history				
Asthma	21 (0.3)	16 (0.3)	5 (0.4)	0.372
Chronic obstructive pulmonary disease	50 (0.7)	36 (0.6)	14 (1.1)	0.034
Pneumonia	117 (1.6)	57 (0.9)	60 (4.9)	<0.001
Atelectasis	23 (0.3)	20 (0.3)	3 (0.2)	1
Pleural effusion	127 (1.7)	95 (1.5)	32 (2.6)	0.011
Cerebral infarction	297 (4.0)	215 (3.5)	82 (6.7)	<0.001
Cerebral hemorrhage	16 (0.2)	13 (0.2)	3 (0.2)	0.737
Diabetes	303 (4.1)	238 (3.8)	65 (5.3)	0.018
Hyperthyroidism	124 (1.7)	101 (1.6)	23 (1.9)	0.54
Hypothyroidism	1,184 (15.9)	1,144 (18.4)	40 (3.3)	<0.001
Liver insufficiency	380 (5.1)	360 (5.8)	20 (1.6)	<0.001
Gastrointestinal bleeding	25 (0.3)	22 (0.4)	3 (0.2)	0.787
Hypertension	865 (11.6)	556 (8.9)	309 (25.3)	<0.001
Hyperlipemia	1,814 (24.4)	1,494 (24.0)	320 (26.2)	0.109
Renal dysfunction	22 (0.3)	17 (0.3)	5 (0.4)	0.39
Coronary artery disease				<0.001
One vessel involved	107 (1.4)	55 (0.9)	52 (4.3)	
Two vessels involved	45 (0.6)	18 (0.3)	27 (2.2)	
Three vessels involved	55 (0.7)	22 (0.4)	33 (2.7)	
Prior endocarditis	191 (2.6)	142 (2.3)	49 (4.0)	0.001
Atrial fibrillation	3,724 (50.0)	3,205 (51.5)	519 (42.5)	<0.001
Peripheral vascular disease	76 (1.0)	57 (0.9)	19 (1.6)	0.06
Congestive heart failure	1,486 (20.0)	1,428 (23.0)	58 (4.8)	<0.001
Prior cardiovascular surgery	1,034 (13.9)	876 (14.1)	158 (12.9)	0.298
NYHA				<0.001
I	123 (1.7)	51 (0.8)	72 (5.9)	
II	1,726 (23.2)	1,234 (19.8)	492 (40.3)	
III	5,332 (71.7)	4,781 (76.9)	551 (45.1)	
IV	260 (3.5)	154 (2.5)	106 (8.7)	
ASA				<0.001
II	270 (3.6)	105 (1.7)	165 (13.5)	
III	6,741 (90.6)	5,774 (92.8)	967 (79.2)	
IV	430 (5.8)	341 (5.5)	89 (7.3)	
Surgery type				
Single-valve	3,347 (45.0)	2,717 (43.7)	630 (51.6)	<0.001
Multi-valve	3,943 (53.0)	3,433 (55.2)	510 (41.8)	<0.001
Combined CABG	151 (2.0)	70 (1.1)	81 (6.6)	<0.001
Intraoperative data				
Operation time, h	4.74 (1.18)	4.87 (1.08)	4.07 (1.41)	<0.001
CPB time, min	118.14 (41.68)	117.65 (38.99)	120.61 (53.26)	0.066
Aortic cross-clamping time, min	79.95 (32.75)	79.42 (31.49)	82.63 (38.45)	0.006
RBC transfusion, U	0.0 (0.0, 0.0)	0.0 (0.0, 0.0)	0.0 (0.0, 3.5)	<0.001
Transfusion of thrombin, U	0.0 (0.0, 2.0)	0.0 (0.0, 2.0)	0.0 (0.0, 0.0)	<0.001
Residual blood in pump after CPB, ml	600 (600, 800)	600 (600, 800)	500 (500, 650)	<0.001

Values are number (proportion), mean (SD) or median (IQR), unless otherwise noted. BMI, body max index; NYHA, New York Heart Association; ASA, American Society of Anesthesiologists; RBC, red blood cell; CABG, coronary artery bypass graft; CPB, cardiopulmonary bypass.

Across all patients at both study sites, the composite of major complications occurred in 200 (2.7%), comprising 86 deaths (1.2%), 9 cases of myocardial infarction (0.1%), 27 cases of stroke (0.4%), and 125 cases of severe acute kidney injury (1.7%; Supplementary Table S3). The development cohort showed significantly lower rates of the composite outcome (129/6,220, 2.1% vs. 71/1,221, 5.8%; *P* < 0.001) as well as significantly lower rates of stroke (0.3% vs. 1.1%, *P* < 0.01) and severe acute kidney injury (0.7% vs. 4.5%, *P* < 0.01). In contrast, rates of mortality and myocardial infarction did not differ significantly between the two cohorts.

### Model development and assessment

**Figure 1:**
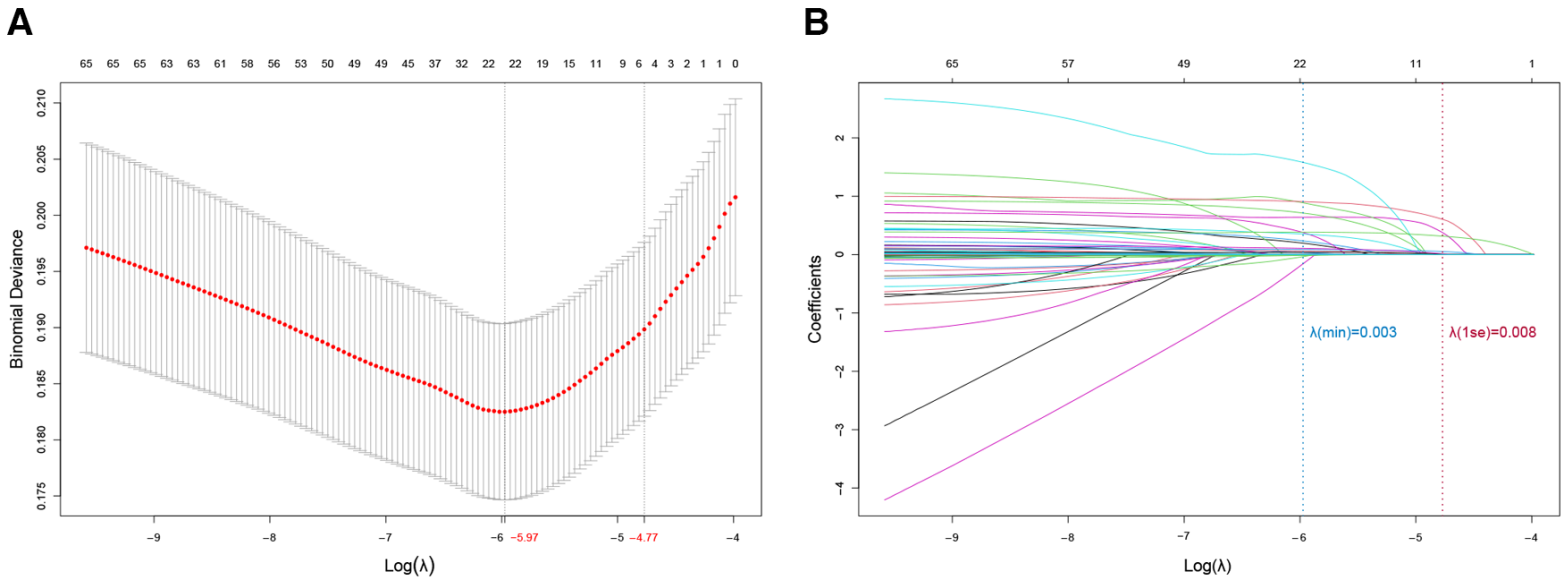
Predictor selection using least absolute shrinkage and selection operator (LASSO) regression. (**A**) Optimization of the penalty tuning parameter *λ* using 10-fold cross-validation with the minimum criterion. The number of predictors is indicated at the top of the plot. Dotted vertical lines indicate the minimum criterion and one standard error away from it. An optimal value of log (*λ*) = −4.82 was selected. (**B**) Coefficient profile plot of the predictors. The number of predictors is indicated at the top of the plot. The ordinate is the coefficient value. Each curve in the figure represents the trajectory of the change of the coefficient of each independent variable, and the coefficient of variable is eventually compressed to 0 as the *λ* parameter increases. Dotted vertical lines were plotted at ideal values, based on the same criteria as in (**A**). Six predictors with non-zero coefficients were selected.

LASSO analysis of 60 variables generated two *λ* parameters, one corresponding to the minimum binomial deviance and the other corresponding to one standard error away from that deviance. We chose the latter *λ* value because it imposed a stricter penalty and could therefore reduce the number of covariates more than the former value. Indeed, increasing *λ* to 0.008, corresponding to one standard error away from the minimum *λ*, led to only six candidate predictors in the logistic model (Figures 1A,B): NYHA class (I, II, III or IV), diabetes (yes or no), blood glucose, blood urea nitrogen (BUN), red blood cell (RBC) transfusion and operation time (Table 2).

**Table 2:** Assessment of potential predictors of the composite outcome after valve surgery based on LASSO regression.

Variable	Odds ratio (95% CI)	*P*-value
NYHA		
I	Reference	
II	1.04 (0.13, 8.41)	0.968
III	1.36 (0.18, 10.52)	0.767
IV	3.98 (0.48, 33.18)	0.201
Diabetes	3.07 (1.72, 5.48)	<0.001
Blood glucose, mmol/L	1.13 (1.01, 1.27)	0.034
BUN, mmol/L	1.09 (1.02, 1.16)	0.007
RBC transfusion, U	1.13 (1.03, 1.23)	0.007
Operation time, h	1.62 (1.44, 1.83)	<0.001

NYHA, New York Heart Association; RBC, red blood cell.

**Figure 2:**
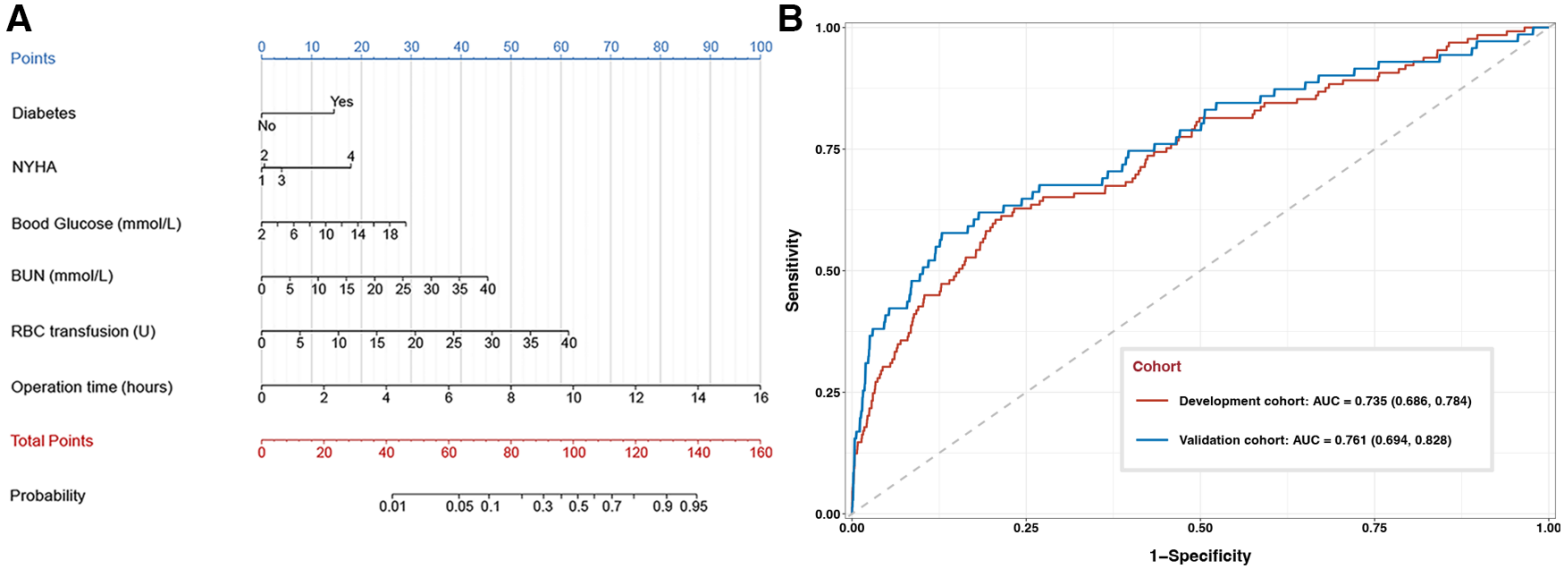
Development and performance assessment of a nomogram to predict composite major complications after valve surgery. (**A**) The nomogram. NYHA, New York Heart Association; BUN, blood urea nitrogen; RBC, red blood cell. (**B**) Receiver operating characteristic curves for the nomogram against the development cohort (red) or validation cohort (blue). The area under the curve (AUC) together with the 95% CI: 0.735 (95% CI, 0.686–0.784) in the development cohort and 0.761 (95% CI, 0.694–0.828) in the validation cohort.

A nomogram was generated based on the model, in which each predictor was scored and the individual scores were summed to obtain the overall probability of the primary outcome (Figure 2A). For example, one patient was diabetic (15 points) and belonged to NYHA class III (4 points). He or she had preoperative levels of blood glucose of 8 mmol/L (10 points) and BUN of 5 mmol/L (6 points). Surgery lasted 4 h (25 points), during which he or she received 5 units of RBC (8 points). The total score for this patient was 68 points, corresponding to 7% composite risk of major complications.

The nomogram discriminated between patients who experienced or not the primary outcome with an AUC of 0.735 (95% CI, 0.686–0.784) in the development cohort and 0.761 (95% CI, 0.694–0.828) in the validation cohort (Figure 2B). When patients were stratified as low- or high-risk based on the optimal cut-off nomogram score of 52.46 points, incidence of the primary outcome was confirmed to be significantly different between low- and high-risk patients, both in the development cohort (1.0% vs. 5.8%) and validation cohort (2.9% vs. 15.8%).

**Figure 3:**
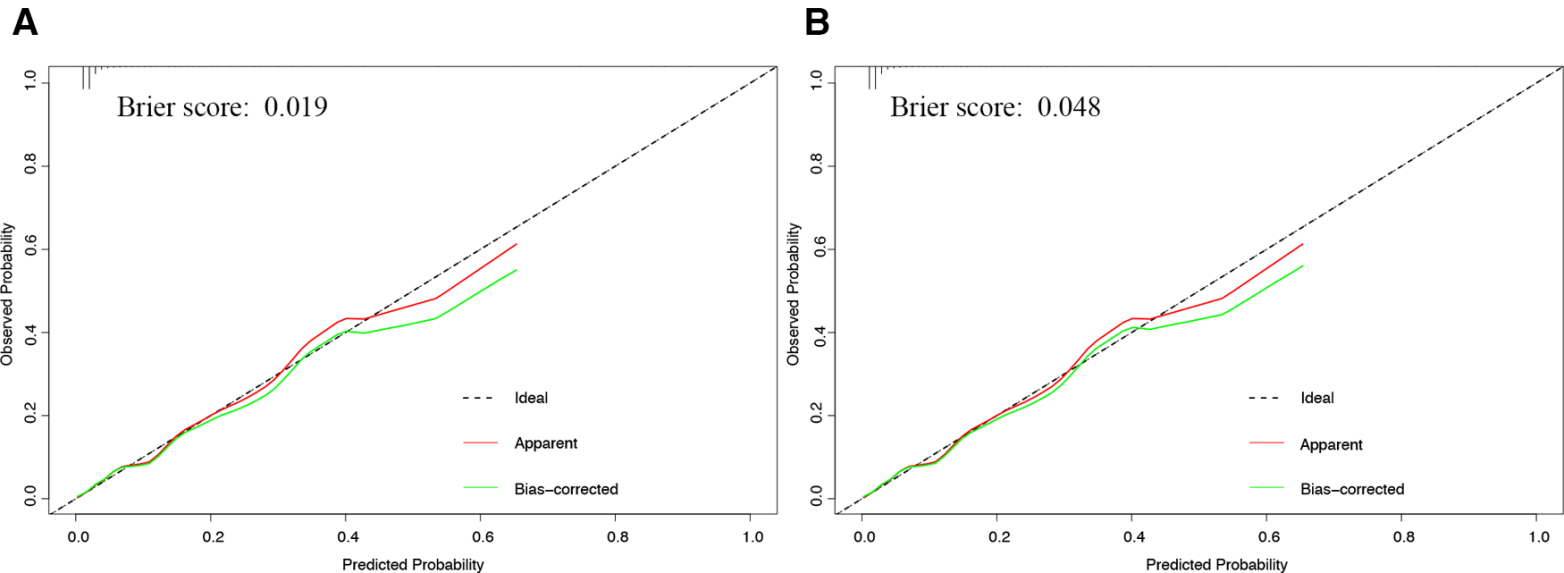
Calibration curve of development and validation cohorts. Calibration curves showing agreement between the nomogram's predicted probability of a composite of major complications and actual probability of the composite in the (**A**) development or (**B**) validation cohort. The dashed diagonal line in the figure indicates when predicted and observed probabilities match perfectly. The red and green fitted curves were apparent and bias-corrected calibration curves respectively. The corresponding Brier scores are shown at the upper left of each plot.

**Figure 4:**
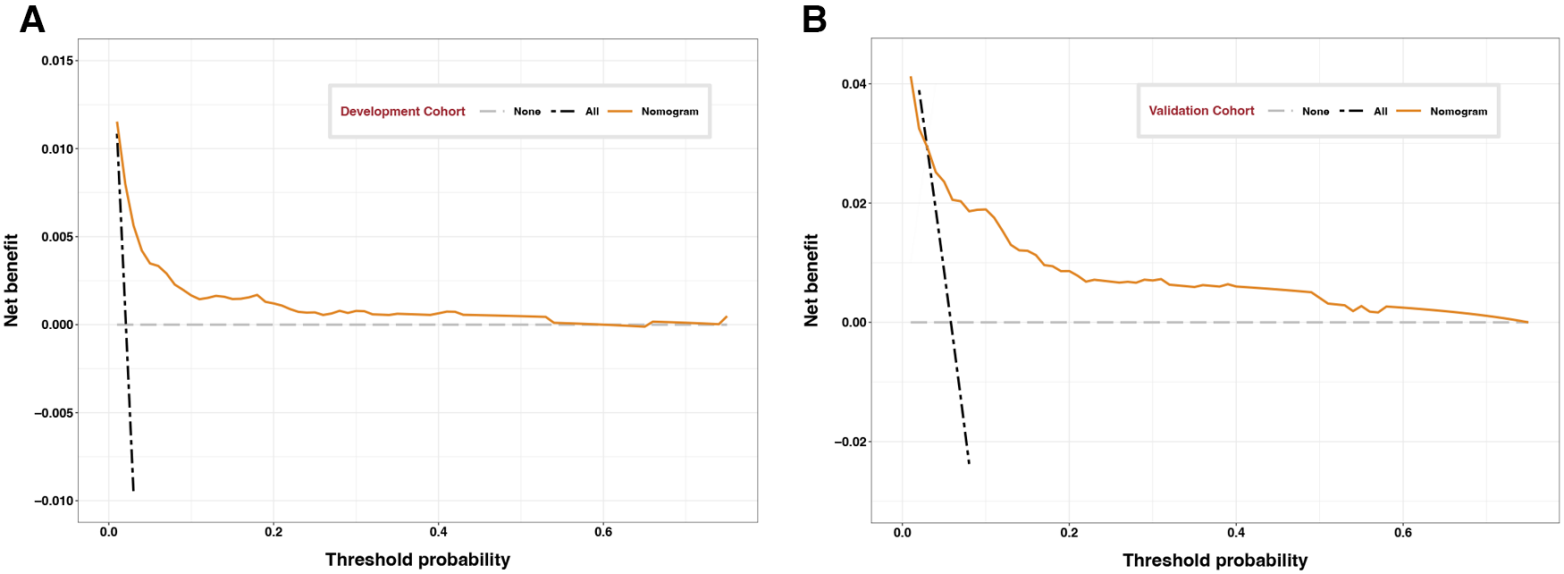
Decision curves of development and validation cohorts. Decision curve analysis assessing the clinical utility of the nomogram for the (**A**) development or (**B**) validation cohort as a function of probability thresholds. Dotted lines represent the states when no patient (black) or all patients (gray) experience the composite outcome of major complications. Clinical benefit is indicated when the orange line lies to the right of the black dotted line and above the gray dotted line.

Calibration curves showed good agreement between observed results and predicted results for the development cohort (Figure 3A) and validation cohort (Figure 3B). The corresponding Brier scores were 0.02 and 0.05, where the ideal score is 0. Decision curve analysis suggested acceptable performance for both the development cohort (Figure 4A) as well as the validation cohort (Figure 4B), although the model performed better for the development cohort.

### Benchmarking of the model against SinoSCORE

SinoSCORE performed worse than our nomogram at predicting the primary outcome, giving AUC 0.597 (95% CI, 0.563–0.631) across all patients, 0.545 (95% CI, 0.508–0.582) in the development cohort and 0.654 (95% CI, 0.591–0.716) in the validation cohort (Supplementary Figure S3). SinoSCORE overestimated the composite risk of major complications in our sample: it predicted rates of 15.8% across all patients, 13.3% in the development cohort and 28.7% in the validation cohort, compared to the corresponding actual rates of 2.7%, 2.1% and 5.8%.

## Discussion

Severe complications, including myocardial infarction, stroke, and severe acute kidney injury strongly compromise prognosis after cardiac surgery (5, 28–30). Using data from more than 7,000 patients at two large tertiary hospitals in China, the present study established a simple risk model based on six easily accessible variables in order to predict composite risk of major complications after valve surgery. To our knowledge, this is the first model that can predict composite risk of major complications after valve surgery. The model may help improve risk screening and management, ultimately leading to better prognosis.

Our model is much simpler than EuroSCORE II (8) or SinoSCORE (9): it includes only four preoperative variables (diabetes, NYHA class, blood glucose and BUN) and two intraoperative variables (intraoperative RBC transfusion and operation time). With only six variables, our nomogram is easier to use. In addition, the predictive variables were all routine test indicators in clinical practice, so information collection would not bring additional burden as time and resources can be saved. It can quickly help to optimize resources and implement prophylactic strategies. The nomogram model is able to identify patients at risk for postoperative major complications immediately after surgery. It may provide “early warning” to medical staff. Patients who are deemed at high risk based on our model may require more careful attention to preoperative blood glucose level and renal function, and they may benefit from individualized perioperative blood management (31) as well as efforts to shorten surgery time as much as possible.

All the variables in our model, except NYHA class, differ from those in the SinoSCORE (9), but are consistent with other reported risk factors of cardiac surgery (16, 32, 33). As reported previously (34), we found that SinoSCORE overestimated the risk of major complications even in patients underwent CABG. This relatively poor performance may reflect that SinoSCORE was designed a decade ago based on data from Chinese patients who underwent CABG. We suggest that SinoSCORE may no longer be suitable for predicting risk of major complications as a result of advances in the surgical treatment and management of cardiac patients.

Surgery type did not substantially influence risk of primary outcome in our sample: it did not survive variable selection in LASSO regression, nor did it substantially alter risk after we manually added it into the regression model along with the six final predictors (Supplementary Table S3). This negative result may reflect that only 70 patients (1.1%) in the development cohort underwent valve replacement with CABG. It may also reflect that patients after most types of complex surgery are generally monitored closely throughout the perioperative period, which may help prevent postoperative complications. Further research with larger samples undergoing different types of cardiac surgery should explore the potential influence of type of surgery on risk of major complications.

Our findings should be interpreted with caution in light of several limitations. The retrospective design meant that we lacked sufficient data to consider some potentially relevant variables, such as preoperative angina or affected mobility (8). Indeed, we benchmarked our model only against SinoSCORE because our database lacked information needed to determine several parameters that appear in EuroSCORE II and other previously published models. Second, there are different incidences of composite outcome in derivation and validation cohorts (2.1% vs. 5.8%), which maybe partly due to different clinical practices and baseline characteristics especially preoperative status. Fortunately, our model shows good discrimination and calibration in both two cohorts. Third, we validated our model with only one external cohort. Given the complexity of cardiac surgery and the therefore large number of perioperative parameters that may differ across medical centers, our results need to be verified and extended through research at additional sites.

## Conclusions

We present a nomogram that may reliably identify patients at high composite risk of major complications after valve surgery. This tool may improve the monitoring and management of this vulnerable patient population.

## Data Availability

The original contributions presented in the study are included in the article/**Supplementary Material**, further inquiries can be directed to the corresponding author.
