# Supplementary material for: A novel model for predicting a composite outcome of major complications after valve surgery
Source: Front Cardiovasc Med. 2023 May 17;10:1132428. doi: 10.3389/fcvm.2023.1132428 (PMC10229809; doi:10.3389/fcvm.2023.1132428)
Supplement: Supplementary file 1 [file Datasheet1.docx]

**Supplementary Files:**

This document has been provided by the authors to give readers additional information about their work.

Supplement to: **A novel model for predicting composite major complications after valve surgery**

**Contents**

| **Appendix Figure/Tables** | **Page** |
| --- | --- |
| - **Appendix Table 1.** Definition of variables and outcomes in the study. | 2 |
| - **Appendix Table 2.** Additional baseline characteristics of patients in the development and external validation cohort. | 8 |
| - **Appendix Table 3.** Comparison of composite major complications incidence and each component between development and external validation cohort. | 10 |
| - **Appendix Table 4.** Logistic regression analysis of variables for predicting adverse events after valve surgery when surgery types were forced into the model. | 11 |
| - **Appendix Figure Titles and Legends.** | 12 |

**Appendix Table 1.** Definition of variables and outcomes in the study.

| **Variable** | **Definition and unit of measurement** |
| --- | --- |
| **Baseline Characteristics** |  |
| Age | Continuous variable (years) |
| Sex | Binomial variable (male/female) |
| Ethnicity | Binomial variable, including Han, Tibetan, and Other |
| Body mass index (BMI) | Continuous variable (kg/m^2^) |
| Current alcohol | Binomial variable (yes/no) |
| Current smoker | Binomial variable (yes/no) |
| **Indications for surgery** | Binomial variable (yes/no). Defined as simple or complex valvular disease, myocardial infarction, aorta-related disease, myxoma, combination of coronary artery disease (CAD) and valvular disease, or infective endocarditis. |
| **Comorbidities or prior disease** | |
| Asthma | Binomial variable (yes/no). Defined as a history of asthma. |
| COPD ^1,2^ | Binomial variable (yes/no). Defined as any of the following conditions: (1) diagnosis retrieved from patient’s history and treatment with bronchodilators and/or corticosteroids; **(**2) incompletely reversible airway obstruction, defined as a ratio of post-bronchodilator forced expiratory volume in 1 s to forced vital capacity (FEV1: FVC) less than 70%. |
| Pneumonia ^3^ | Binomial variable (yes/no). Defined as the presence of infiltrate on a chest radiograph combined with temperature over 38 °C and elevated leukocytosis. |
| Atelectasis ^3^ | Binomial variable (yes/no). Defined as lung opaciﬁcation with a shift of the mediastinum, hilum, or hemidiaphragm toward the affected area, and compensatory over-inﬂation in the adjacent non-atelectatic lung. |
| Hydrothorax | Binomial variable (yes/no). Defined as an examination indicating hydrothorax. |
| Cerebral infarction | Binomial variable (yes/no). Defined as a history of cerebral infarction. |
| Cerebral hemorrhage ^4^ | Binomial variable (yes/no). Defined as examination indicating hemorrhage, and an acute episode of neurological dysfunction caused by hemorrhage-induced focal or global injury in the brain, spinal cord, or retinal vasculature. |
| Diabetes ^4^ | Binomial variable (yes/no). Defined as a history of diabetes diagnosed and/or treated by a physician. The American Diabetes Association criteria include documentation of the following: (1) hemoglobin A1c > 6.5%; (2) fasting plasma glucose ≥ 126 mg/dL (7.0 mmol/L); (3) two-hour plasma glucose ≥ 200 mg/dL (11.1 mmol/L) during an oral glucose tolerance test; or (4) randomly tested plasma glucose >200 mg/dL (11.1 mmol/L) in patients with classic symptoms of hyperglycemia or hyperglycemic crisis. |
| Hyper- or Hypothyroidism | Binomial variable (yes/no). Defined as a history of hyper- or hypothyroidism. |
| Liver insufficiency ^5^ | Binomial variable (yes/no). Defined as an increase of more than two times the upper limit of the normal range (2N) in serum alanine aminotransferase (ALT) or conjugated bilirubin, or combined increases in aspartate aminotransferase (AST), alkaline phosphatase (AP), and total bilirubin, as long as one of them exceeds 2N. |
| Renal dysfunction ^6^ | Binomial variable (yes/no). Defined as serum creatinine before surgery greater than 176 μmol/L or a requirement for renal replacement therapy. |
| Gastrointestinal bleeding ^2^ | Binomial variable (yes/no). Based on medical records, and including occult gastrointestinal bleeding, which was defined in accordance with the American Gastroenterological Association as initial presentation of a positive fecal occult blood test and/or iron-deficiency anemia in the absence of visible blood loss. |
| Hypertension ^4^ | Binomial variable (yes/no). Defined as current or previous diagnosis of hypertension based on any of the following: (1) history of hypertension diagnosed and treated with medication, diet and/or exercise; (2) on at least 2 occasions, documented blood pressure > 140 mmHg (systolic) and/or 90 mmHg (diastolic) in patients without diabetes or chronic kidney disease, or > 130 mmHg (systolic) or 80 mmHg (diastolic) in patients with diabetes or chronic kidney disease; or (3) current pharmacological therapy for treatment of hypertension |
| Hyperlipemia | Binomial variable (yes/no). Defined as an examination indicating, or a history of, hyperlipemia. |
| CAD ^7^ | Binomial variable (yes/no). Recorded as the number of stenoses in the coronary artery. If details of coronary artery were unavailable, the result was recorded as “not investigated”. |
| Prior endocarditis ^4^ | Binomial variable (yes/no). Defined according to the Duke criteria. |
| Atrial Fibrillation | Binomial variable (yes/no). Defined as a history of atrial fibrillation. |
| Peripheral vascular disease ^4^ | Binomial variable (yes/no). Defined as a history of lower extremity peripheral arterial disease (from iliac to tibial), excluding renal, coronary, cerebral, or mesenteric vessels or aneurysm. Major symptoms could include: (1) asymptomatic, confirmed by noninvasive diagnostic testing; (2) claudication relieved by rest; (3) ischemic pain at rest; (4) tissue loss, including ischemic ulcer and/or gangrene; (5) amputation for critical limb ischemia; (6) vascular reconstruction, bypass surgery, or percutaneous revascularization in the arteries of the lower extremities; or (7) positive noninvasive test, such as ankle-brachial index ≤ 0.90, imaging (ultrasonography, magnetic resonance, or computed tomography) demonstrating > 50% diameter stenosis in any peripheral artery (aorta, iliac, femoral, popliteal, tibial, peroneal). |
| Congestive heart failure (CHF) ^4^ | Binomial variable (yes/no). Defined as a history of CHF, including previous hospital admission with a principal diagnosis of CHF. CHF was defined as documentation of any two of the following Framingham major criteria of heart failure: orthopnea/paroxysmal nocturnal dyspnea; or the description of rales, jugular venous distention, hepatojugular reflux, S3 gallop, or pulmonary edema on chest x-ray; or one of the major criteria plus two Framingham minor criteria, including dyspnea on exertion, nocturnal cough, ankle edema, pleural effusion, or tachycardia. A low ejection fraction without clinical evidence of heart failure did not qualify as heart failure. |
| Prior cardiovascular surgery | Binomial variable (yes/no). Defined as open cardiovascular surgery or PCI, such as balloon angioplasty, atherectomy, stent, or other. |
| **NYHA classification** ^8^ | Ordinal categorical variable |
| I | Cardiac disease, but no symptoms and no limitation of ordinary physical activity, e.g. no shortness of breath when walking or climbing stairs. |
| II | Mild symptoms (mild shortness of breath and/or angina) and slight limitation during ordinary activity. |
| III | Marked limitation in activity due to symptoms, even during less-than-ordinary activity, e.g. walking short distances (20-100 m), comfortable only at rest. |
| IV | Severe limitations, symptoms even while at rest. Mostly bedridden patients. |
| **ASA physical status** ^9^ |  |
| I | A normal healthy patient. |
| II | A patient with mild systemic disease. |
| III | A patient with severe systemic disease. |
| IV | A patient with severe systemic disease that poses a constant threat to life. |
| V | A moribund patient who is not expected to survive without the operation. |
| VI | A declared brain-dead patient whose organs are being removed for donor purposes. |
| **Medications** | Binomial variable (yes/no). Defined as being on medication at the time of surgery. Medications include β-adrenergic receptor blockers, clopidogrel, aspirin, cardiotonics, anticoagulants, or diuretics. |
| **Laboratory findings** | Data were extracted from electronic medical records. For pulmonary hypertension, this variable was recorded as an ordinal categorical one: “mild” referred to mean pulmonary artery pressure, 25-34 mmHg; “moderate”, 35-44 mmHg; or “severe”, ≥45 mmHg. |
| **Type of surgery** | Single valve replacement, multiple valve replacement, or valve surgery combined with coronary artery bypass graft |
| **Intraoperative data** |  |
| Operation time | Categorical variable (hours). |
| Cardiopulmonary bypass duration (CPB) | Continuous variable (min). Defined as the time from start to end of CPB. If the patient underwent the procedure more than once, the CPB duration was defined as the sum of the procedures. |
| Aortic cross-clamping time | Continuous variable (min). Defined as the time from start to end of aortic cross-clamping. If the patient underwent the procedure more than once, the aortic cross-clamping duration was defined as the sum of the procedures. |
| Transfusion | Data were extracted from electronic medical records, including red blood cell, thrombin, or residual blood in the pump after CPB. |
| **Outcome** |  |
| All-cause mortality ^1^ | Death due to any cause occurring at any time from the end of surgery until hospital discharge |
| Myocardial infarction ^1^ | Either the occurrence of new Q waves on the electrocardiogram or ischemic ST changes in combination with abnormal postoperative troponin T levels (troponin T level > 0.5 μg/L for coronary artery bypass graft surgery, troponin T level > 0.8 μg/L for valve surgery, or troponin T level > 1.0 μg/L for the combination of valve procedures and coronary artery bypass graft). |
| Stroke ^7^ | Documentation of computed tomography or magnetic resonance imaging indicating stroke as well as documentation of new-onset focal or generalized neurological deficit, defined as a deficit in motor, sensory or coordination functions. |
| Severe acute kidney injury (AKI) ^10^ | Stage 3 of AKI: serum creatinine >3-fold higher than the preoperative value, or serum creatinine ≥4.0 mg/dL (≥354 μmol/L) with an acute increase of at least 0.5 mg/dL (44 μmol/L), or urine output <0.3 ml/kg for 24 hours or anuria for 12 hours, or need for renal replacement therapy (RRT) irrespective of AKI stage at the time of RRT. |

**References**

1. Hogervorst E, Rosseel P, van der Bom J, Bentala M, Brand A, van der Meer N, et al. Tolerance of intraoperative hemoglobin decrease during cardiac surgery. *Transfusion.* 2014;54(10 Pt 2):2696-2704.doi:10.1111/trf.12654.
2. Postma DS, Bush A, van den Berge M. Risk factors and early origins of chronic obstructive pulmonary disease. *Lancet.* 2015;385(9971):899-909.doi:10.1016/S0140-6736(14)60446-3.
3. Jammer I, Wickboldt N, Sander M, Smith A, Schultz MJ, Pelosi P, et al. Standards for definitions and use of outcome measures for clinical effectiveness research in perioperative medicine: European Perioperative Clinical Outcome (EPCO) definitions: a statement from the ESA-ESICM joint taskforce on perioperative outcome measures. *Eur J Anaesthesiol.* 2015;32(2):88-105.doi:10.1097/EJA.0000000000000118.
4. Creager MA, Belkin M, Bluth EI, Casey DE, Jr., Chaturvedi S, Dake MD, et al. 2012 ACCF/AHA/ACR/SCAI/SIR/STS/SVM/SVN/SVS Key data elements and definitions for peripheral atherosclerotic vascular disease: a report of the American College of Cardiology Foundation/American Heart Association Task Force on Clinical Data Standards (Writing Committee to develop Clinical Data Standards for peripheral atherosclerotic vascular disease). *J Am Coll Cardiol.* 2012;59(3):294-357.doi:10.1016/j.jacc.2011.10.860.
5. Raju GS, Gerson L, Das A, Lewis B, American Gastroenterological A. American Gastroenterological Association (AGA) Institute medical position statement on obscure gastrointestinal bleeding. *Gastroenterology.* 2007;133(5):1694-1696.doi:10.1053/j.gastro.2007.06.008.
6. Oida K, Takai H, Maeda H, Takahashi S, Shimada A, Suzuki J, et al. Apolipoprotein(a) is present in urine and its excretion is decreased in patients with renal failure. *Clin Chem.* 1992;38(11):2244-2248
7. Murphy GJ, Pike K, Rogers CA, Wordsworth S, Stokes EA, Angelini GD, et al. Liberal or restrictive transfusion after cardiac surgery. *N Engl J Med.* 2015;372(11):997-1008.doi:10.1056/NEJMoa1403612.
8. The Criteria Committee of the New York Heart Association. (1994). Nomenclature and Criteria for Diagnosis of Diseases of the Heart and Great Vessels (9th ed.). Boston: Little, Brown & Co. pp. 253–256.
9. Hurwitz EE, Simon M, Vinta SR, Zehm CF, Shabot SM, Minhajuddin A, et al. Adding Examples to the ASA-Physical Status Classification Improves Correct Assignment to Patients. *Anesthesiology.* 2017;126(4):614-622.doi:10.1097/ALN.0000000000001541.
10. Schneider A, Ostermann M. The AKI glossary. *Intensive Care Med.* 2017;43(6):893-897.doi:10.1007/s00134-017-4751-y.

**Appendix Table 2.** Additional baseline characteristics of patients in the development and validation cohorts.

| **Variable** | **Overall**  **(n = 7441)** | **Development cohort**  **(n= 6220)** | **Validation cohort**  **(n = 1221)** | ***P* value** |
| --- | --- | --- | --- | --- |
| **Medications** |  |  |  |  |
| β-adrenergic receptor blocker | 744 (10.0) | 606 (9.7) | 138 (11.3) | 0.106 |
| Clopidogrel | 65 (0.9) | 9 (0.1) | 56 (4.6) | <0.001 |
| Aspirin | 127 (1.7) | 13 (0.2) | 114 (9.3) | <0.001 |
| Cardiotonics | 2330 (31.3) | 2193 (35.3) | 137 (11.2) | <0.001 |
| Anticoagulants | 865 (11.6) | 549 (8.8) | 316 (25.9) | <0.001 |
| Diuretics | 5574 (74.9) | 5334 (85.8) | 240 (19.7) | <0.001 |
| **Laboratory findings** |  |  |  |  |
| LVEF, % | 60.64 (9.15) | 60.85 (9.14) | 59.61 (9.09) | <0.001 |
| Pulmonary hypertension |  |  |  | <0.001 |
| Mild | 446 (6.0) | 266 (4.3) | 180 (14.7) |  |
| Moderate | 759 (10.2) | 588 (9.5) | 171 (14.0) |  |
| Severe | 352 (4.7) | 288 (4.6) | 64 (5.2) |  |
| Hemoglobin, g/dL | 13.43 (1.79) | 13.49 (1.77) | 13.13 (1.90) | <0.001 |
| RBC count, *10^12/L | 4.54 (0.61) | 4.57 (0.59) | 4.40 (0.68) | <0.001 |
| WBC count, *10^9/L | 5.79 (4.83, 6.97) | 5.78 (4.85, 6.93) | 5.80 (4.70, 7.20) | 0.411 |
| PLT count, *10^9/L | 150 (115, 192) | 148 (113, 188) | 166 (128, 207) | <0.001 |
| PT, s | 12.10 (11.40, 13.10) | 11.90 (11.30, 12.70) | 13.50 (12.90, 14.70) | <0.001 |
| APTT, s | 29.80 (26.60, 34.30) | 28.80 (26.10, 31.80) | 38.20 (35.00, 42.40) | <0.001 |
| Fibrinogen, g/L | 2.77 (2.36, 3.28) | 2.71 (2.33, 3.20) | 3.07 (2.60, 3.57) | <0.001 |
| INR | 1.05 (0.99, 1.12) | 1.05 (0.99, 1.12) | 1.05 (0.99, 1.17) | 0.048 |
| Albumin, g/L | 42.30 (39.60, 44.90) | 42.70 (40.20, 45.20) | 40.00 (37.40, 42.80) | <0.001 |
| ALT, IU.L^-1^ | 21 (15, 31) | 22 (16, 31) | 19 (14, 29) | <0.001 |
| Total bilirubin, μmol/L | 15.3 (11.4, 20.9) | 15.5 (11.5, 21.1) | 14.4 (11.2, 19.7) | 0.006 |
| BUN, mmol/L | 5.70 (4.60, 6.98) | 5.64 (4.60, 6.90) | 5.87 (4.80, 7.26) | <0.001 |
| Scr, μmol/L | 70.5 (61.0, 82.0) | 71.1 (62.0, 82.5) | 65.4 (55.0, 79.0) | <0.001 |
| Cys-C, mg/L | 1.04 (0.93, 1.19) | 1.03 (0.92, 1.16) | 1.13 (0.98, 1.34) | <0.001 |
| Blood glucose; mmol/L | 4.91 (4.56, 5.35) | 4.91 (4.56, 5.35) | 4.88 (4.55, 5.37) | 0.657 |

Values are mean (SD) or median (IQR) for continuous variables and number (proportion) for categoric variables

LVEF, left ventricular ejection fraction; RBC, Red blood cell; WBC, white blood cell; PLT, platelet; PT, prothrombin time; APTT, activated partial thromboplastin time; INR, international normalized ratio; ALT, alanine aminotransferase; BUN, blood urea nitrogen; Scr, serum creatinine; Cys-C, Cystatin C.

**Appendix Table 3.** Incidence of the composite outcome and major individual complications in the development and validation cohorts.

| **Outcome** | **Overall**  **(n = 7441)** | **Development cohort**  **(n = 6220)** | **Validation cohort**  **(n = 1221)** | ***P*-value** |
| --- | --- | --- | --- | --- |

| **Composite major complications** | 200 (2.7) | 129 (2.1) | 71 (5.8) | <0.001 |
| --- | --- | --- | --- | --- |
| Mortality | 86 (1.2) | 71 (1.1) | 15 (1.2) | 0.77 |
| Myocardial infarction | 9 (0.1) | 8 (0.1) | 1 (0.1) | 1 |
| Stroke | 27 (0.5) | 18 (0.3) | 9 (0.7) | 0.032 |
| Severe acute kidney injury | 125 (1.7) | 70 (1.1) | 55 (4.5) | <0.001 |

Values are number (proportion) unless otherwise noted.

**Appendix Table 4.** Logistic regression to identify potential predictors using a model that took into account surgery type.

| **Variable** | **OR (95%CI)** | ***P*-value** |
| --- | --- | --- |
| Diabetes | 3.17 (1.77, 5.66) | <0.001 |
| NYHA I | (Reference) |  |
| NYHA II | 0.98 (0.12, 7.81) | 0.981 |
| NYHA III | 1.31 (0.17, 10.06) | 0.794 |
| NYHA IV | 3.86 (0.47, 32) | 0.210 |
| Blood glucose (mmol/L) | 1.14 (1.01, 1.28) | 0.029 |
| BUN (mmol/L) | 1.09 (1.02, 1.16) | 0.008 |
| Transfusion of RBC (U) | 1.13 (1.03, 1.23) | 0.007 |
| Single valve surgery | (Reference) |  |
| Multiple valve surgery | 0.85 (0.58, 1.24) | 0.397 |
| Combined CABG | 0.57 (0.17, 3.94) | 0.368 |

NYHA, New York Heart Association; BUN, blood urea nitrogen; RBC, red blood cell;

CABG, Coronary Artery Bypass Graft.

**Appendix Figure 1.** Flowchart of study design.

CPB, cardiopulmonary bypass; SAHZU, Second Affiliated Hospital of Zhejiang University

**Appendix Figure 2.** Visual presentation of proportions and patterns of missing data for different variables.
**(A)** Categorical variables in the development cohort. **(B)** Categorical variables in the external validation cohort. **(C)** Continuous variables in the development cohort. **(D)** Continuous variables in the external validation cohort.

Histograms on the *left* show the proportions of patients for whom data for the indicated variable were missing. The patterns on the *right* indicate variables for which data were missing (gray) or complete (blue). The histogram on the *far right* indicates the ratio of patients with complete data to patients with missing data for the variable. Abbreviations in this figure are defined in Table1 and Appendix Table2.

**Appendix Figure 3.** Receiver operating characteristic curves to assess the predictive ability of SinoSCORE.

The ability to discriminate patients who did or did not experience the composite outcome of major complications. Total cohort; red, development cohort; blue and validation cohort; green. The area under the curve (AUC) is also indicated together with the 95% CI: 0.597 (95% CI, 0.563–0.631) across all patients, 0.545 (95% CI, 0.508–0.582) in the development cohort and 0.654 (95% CI, 0.591–0.716) in the validation cohort.
